# Regulation of matriptase and HAI-1 system, a novel therapeutic target in human endometrial cancer cells

**DOI:** 10.18632/oncotarget.23913

**Published:** 2018-01-03

**Authors:** Pengming Sun, Lifang Xue, Yiyi Song, Xiaodan Mao, Lili Chen, Binhua Dong, Elena Loana Braicu, Jalid Sehouli

**Affiliations:** ^1^ Laboratory of Gynecologic Oncology, Fujian Provincial Maternity and Children Hospital, Affiliate Hospital of Fujian Medical University, 350001 Fuzhou, Fujian, P.R. of China; ^2^ Department of Gynecology, Fujian Provincial Maternity and Children Hospital, Affiliate Hospital of Fujian Medical University, 350001 Fuzhou, Fujian, P.R. of China; ^3^ Department of Gynecologic Oncology and Gynecology, Charité, Campus Virchow-Klinikum, European Competence Center for Ovarian Cancer University of Berlin, 13353 Berlin, Germany

**Keywords:** matriptase, HAI-1, endometrial cancer, target therapy, cisplatin

## Abstract

The effects of specific and non-specific regulation of matriptase on endometrial cancer cells *in vitro* were investigated. Messenger ribonucleic acid (mRNA) and protein expression of matriptase and hepatocyte growth factor activator inhibitor-1 (HAI-1) in RL-952, HEC-1A, and HEC-1B endometrial cancer cells were detected by real-time quantitative PCR (RT-qPCR) and western blot. The cells were infected with lentivirus-mediated small-interfering RNA (siRNA) targeted on matriptase (MA-siRNA) or treated with different cisplatin (DDP) concentrations. After treatment, invasion, migration, and cellular apoptosis were analyzed. Matriptase mRNA and protein expression significantly decreased to 80% after infection with MA-siRNA (*P* < 0.01), and scratch and trans-well chamber assays showed significant inhibition of invasiveness and metastasis. Upon incubation with cisplatin at concentrations higher than the therapeutic dose for 24 h, the expressions of matriptase and HAI-1 significantly decreased (*P* < 0.001). Moreover, the invasiveness, metastasis, and survival rate of HEC-1A and RL-952 endometrial cancer cells were significantly decreased (*P* < 0.001) due to the down-regulation of matriptase and HAI-1 upon increasing cisplatin concentration. However, a slight increase in matriptase and HAI-1 expression was observed in cells treated with low cisplatin concentration (*P* = 0.01). Moreover, matriptase expression was associated with metastasis and invasiveness. Down-regulation of matriptase by specific Ma-SiRNA or non-specific cisplatin in matriptase/HAI-1–positive endometrial cancer cells showed promising therapeutic features.

## INTRODUCTION

Endometrial cancer is one of the most common malignancies of the female reproductive tract, and its incidence is currently increasing. The American Cancer Society estimated there would be 60,050 new cases and 10,470 deaths from endometrial cancer in 2016 [1]. Asian nations such as China, Japan, and Korean have lower incidence than do western industrialized countries. However, the incidence of endometrial cancer in China has increased over the past 30 years, and it is currently the second most common gynecologic malignancy [2]. Although most of endometrial cancer patients are diagnosed early due to irregular uterine bleeding and abnormal vaginal discharge or other symptoms, there are still 15–25% of patients at advanced stage [3]. Moreover, patients with either advanced stage endometrial cancer at diagnosis or recurrent disease present a considerable therapeutic challenge [4]. Advanced-stage endometrial cancer seriously threatens the patient's health and is responsible for most deaths [4]. Optimal treatment approaches yield response rates of 40–70% in patients with primary advanced cancer and 15–30% in patients with recurrent disease. Furthermore, among these patients, median progression-free survival is only 6 months and median overall survival is 12 months [5]. For the advanced endometrial cancer, metastases are the major cause of treatment failures and mortality. Thus, studies on the invasive and metastatic mechanism are essential to improve advanced endometrial cancer-related survival and cure rate.

Tumor invasion and metastasis formation are complex biological processes depending on the matrix-degrading proteolytic system, which allows tumor cells to detach from their primary site and migrate to distant sites. Matriptase is a type-II transmembrane serine protease (TTSP) of about 855 amino acids and belongs to the family of S1 trypsin-like protease [6, 7]. It combines an amino-terminal hydrophobic transmembrane region with an extracellular section of several domains including trypsin-like catalytic and low-density lipoprotein regions. Moreover, matriptase is expressed not only in epithelial cells, but also in mast cells, B-cells, and blood monocytes [8, 9, 10]. The expression levels of matriptase reflect the degree of tumor progression in several types of cancerous cells, which indicates the crucial role of this protein in malignant cells metastasis [11, 12, 13]. Moreover, matriptase is implicated in a number of other diseases and induces cancer itself [13]. Therefore, matriptase has become a promising target for anti-cancer treatments. Interestingly, matriptase is controlled by its endogenous inhibitor HAI-1 (Hepatocyte growth factor activator inhibitors-1). It has been reported that matriptase and HAI-1 are closely related to the development and progression of many malignant tumors such as esophageal cancer, breast cancer, and prostate cancer [14, 15, 16] and have strong potential oncogenic effect, which may influence tumor invasion and metastasis [17]. However, only two studies about the expression of matriptase and HAI in endometrial cancer have been published on the PUBMED until now [18, 19]. The role of matriptase in endometrial cancer remains unclear. In this study, we analyzed the expression of matriptase and HAI-1 in endometrial cancer cell lines and analyzed their relationship with the invasion and migration of endometrial cancer cells.

## RESULTS

### Expression of matriptase and HAI-1 in endometrial cancer cells

**Figure 1:**
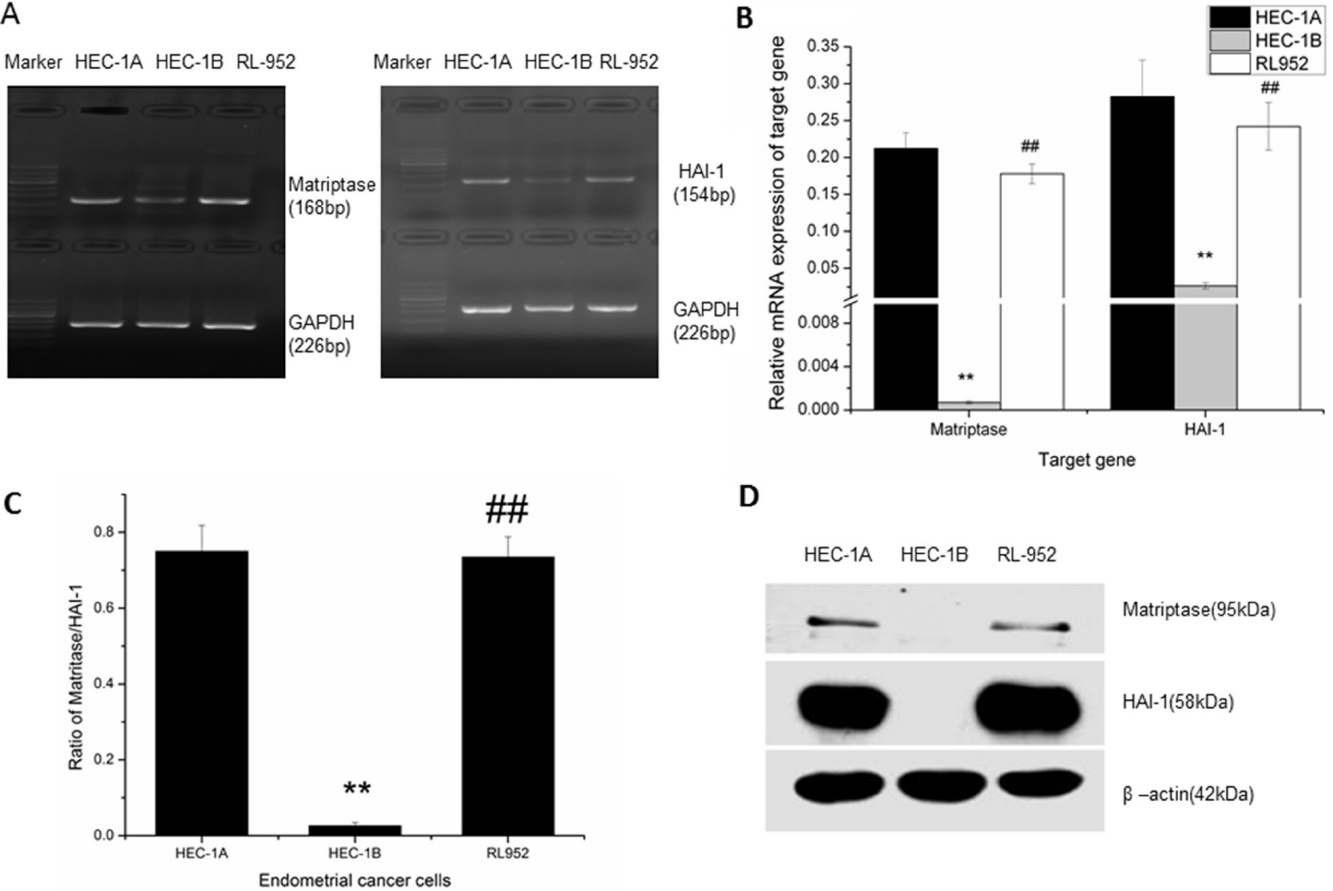
Expression of matriptase and HAI-1 in HEC-1A, HEC-1B, and RL-952 endometrial cancer cells (**A**) mRNA expression of matriptase (168 bp) and HAI (154 bp) detected by PCR in endometrial cancer cells. (**B**) detected by quantitative-PCR, related mRNA expression pattern of matriptase and HAI-1 in endometrial cancer cells. High expression of matriptase and HAI-1 were detected in HEC-1A and RL-952 cells, while it was almost negative expression of matriptase and HAI-1 in HEC-1B cell. (**C**) mRNA expression ratio of matriptase /HAI in endometrial cancer cells. (**D**) protein expression pattern of matriptase and HA-1 in endometrial cells. ^**^Compared to HEC-1A cell, a significant low expression of matriptase and HAI-1 were detected in HEC-1B cell, and ^##^there is no significant difference with RL-952 cell.

The mRNA expression of matriptase and HAI-1 was detected by The levels of matriptase and HAI-1 mRNA expression in HEC-1A, HEC-1B, and RL-952 endometrial cancer cell lines were determined using quantitative PCR. The relative mRNA expression of matriptase was 0.212 ± 0.021 in HEC-1A and 0.178 ± 0.013 in RL-952, while it was only 0.00695 ± 0.0012 in HEC-1B (F = 122.629, *P* < 0.001) (Figure 1A). The relative mRNA expression of HAI-1 was 0.283 ± 0.049 in HEC-1A and 0.242 ± 0.032 in RL-952. Similar to matriptase mRNA expression, HAI-1 mRNA expression was only 0.0263 ± 0.0043 in HEC-1B (F = 32.875, *P* < 0.01) (Figure 1A). The matriptase/HAI-1 mRNA expression ratio in HEC-1A, RL-952, and HEC-1B was 0.75, 0.73, and 0.02, respectively (Figure 1B). The western blot showed the same protein expression pattern (Figure 1C). Both matriptase and HAI could only be detected extreme weak mRNA expression in the HEC-1B cells. Thus, we concluded that, in HEC-1B cells, the matriptase/HAI signal pathway may play a different role with the other two cells.

### Down-regulation of matriptase by lentivirus-mediated small interfering RNA

**Figure 2:**
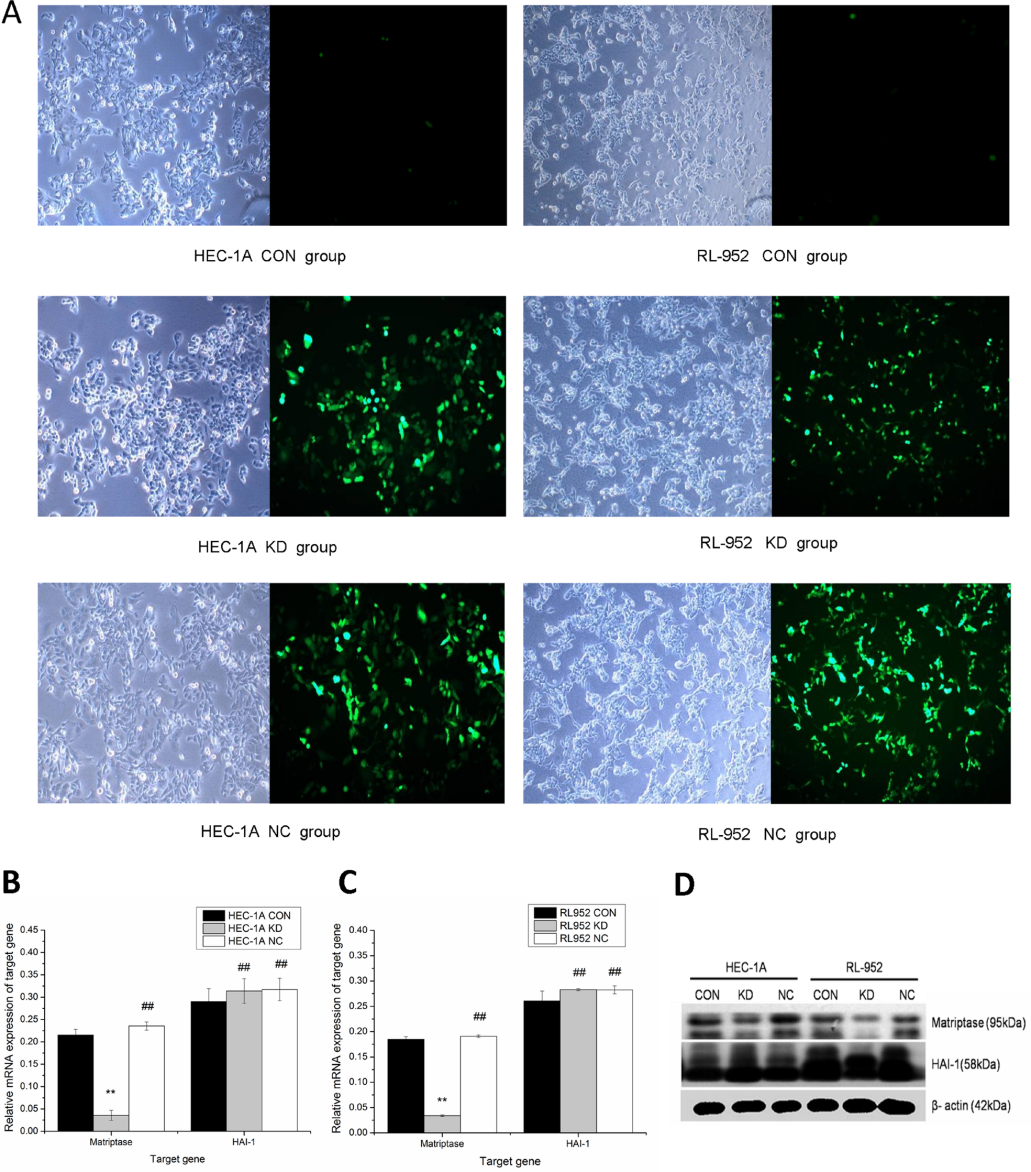
Down regulation of matriptase mediated by Lentivirus siRNA infection (**A**) Infection with lentivirus mediated siRNA targeted on matriptase, HEC-1A and RL-952 cells were observed by optical microscope and fluorescence microscopic with a magnification of 400. Matriptase mRNA expression was significantly down-regulated after the infection with siRNA in HEC-1A (**B**) and RL-952 cell (**C**). (**D**) The protein levels of matriptase and HAI-1 show consistent situation with expression of mRNA and after the infection with lentivirus. ^**^mean *P* < 0.05, ^##^mean *P* > 0.05.

The lentivirus-mediated siRNA plasmid targeting on matriptase was constructed and used to infect the cells (Figure 2A). Compared with the CON group, matriptase mRNA expression of the KD group was significantly down-regulated in both HEC-1A (CON, 0.2157 ± 0.0124; KD, 0.0358 ± 0.0111) and in RL-952 cells (CON, 0.1849 ± 0.0053; KD, 0.0341 ± 0.0017; *P* < 0.01). The inhibition rates were 83.4% and 81.5% in HEC-1A and RL-952, respectively. There was no difference in the expression level of matriptase between the NC and CON groups. HAI-1 mRNA expression levels in KD and NC groups slightly increased compared to that in the CON group in both HEC-1A (KD, 0.3142 ± 0.0277; NC, 0.3175 ± 0.0251; CON 0.2900 ± 0.0292) and RL-952 (KD, 0.2829 ± 0.0021; NC, 0.2826 ± 0.0080; CON, 0.2611 ± 0.0192). However, the increase was not statistically significant (P > 0.05) (Figure 2B–2C). The mRNA and protein levels of matriptase and HAI-1 showed a similar expression pattern (Figure 2B–2D).

### Inhibition of migration and invasion ability in endometrial cancer cells by down-regulation of matriptase

**Figure 3:**
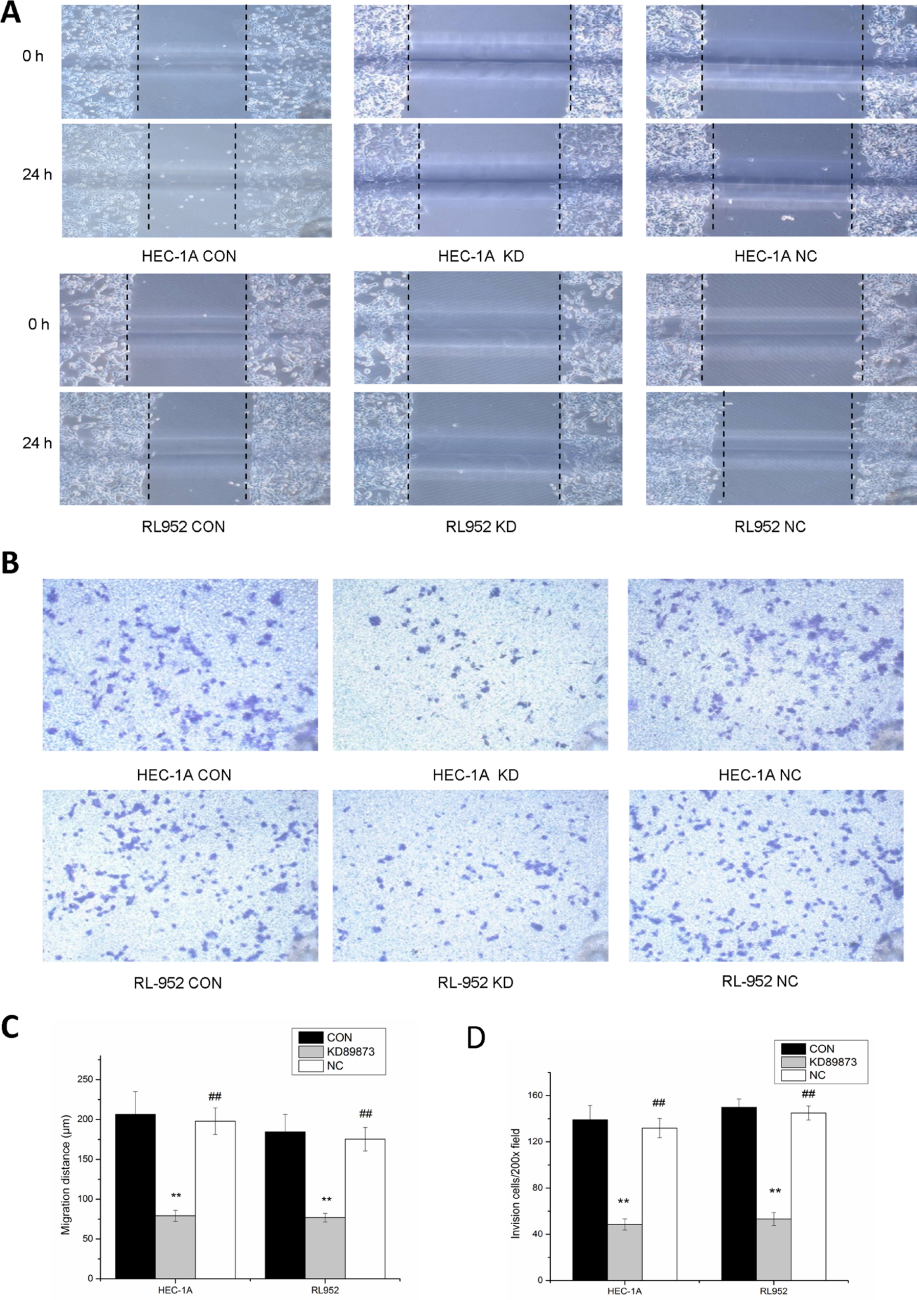
Inhibition of the migration and invasion ability of endometrial cancer cells by down-regulation of matriptase with siRNA (**A**) Compared with blank control groups (CON) and negative control groups (NC), the 24-hour migrated distances of HEC-1A-KD and RL-952-KD group were significantly reduced. (**B**) After incubation for 24 h, compared with CON group and NC group, the endometrial cancer cells penetrated the Transwell membrane were significantly reduced in KD group. Quantification of migration (**C**) and invasion (**D**) of HEC-1A and RL-952 cells. ^**^mean *P* < 0.05, ^##^mean *P* > 0.05.

Migration distances at 24 h were significantly longer in the HEC-1A and RL-952 CON groups than they were in the HEC-1A KD group (CON, 206.67 ± 28.38; KD, 79.2 ± 6.82, *P* < 0.001) and RL-952 KD group (CON, 184.57 ± 21.97; KD, 76.8 ± 5.48; *P* < 0.001) (Figure 3A–3C). The NC group showed results similarly to those of the CON group in both HEC-1A and RL-952 cells (*P* > 0.05). Person's correlation analysis showed that matriptase mRNA expression level was positively correlated with the migration distance with *r* = 0.97 and *r* = 0.982 in HEC-1 and RL-952, respectively. Compared with the CON group, the transmembrane cell number in the KD group was significantly reduced in both HEC-1A cells (CON, 139.25 ± 12.3112; KD, 48.6 ± 4.8496) and in RL-952 cells (CON, 150 ± 7.0710; KD, 53.3 ± 5.6376, *P* < 0.05). Again, the NC group gave results similarly to those of the CON group. Compared to the control group, the NC gave similar result in both HEC-1A (132 ± 8.3120) and RL-952 cells (145 ± 6.0711, *P* > 0.05) (Figure 3B, 3D). Pearson's correlation analysis showed positive correlation between matriptase mRNA expression levels and transmembrane cell number with *r* = 0.975 and *r* = 0.994 in HEC-1A and RL-952, respectively.

### Cisplatin dose- and time-dependent regulation of matriptase and HAI-1 in endometrial cancer cells

**Figure 4:**
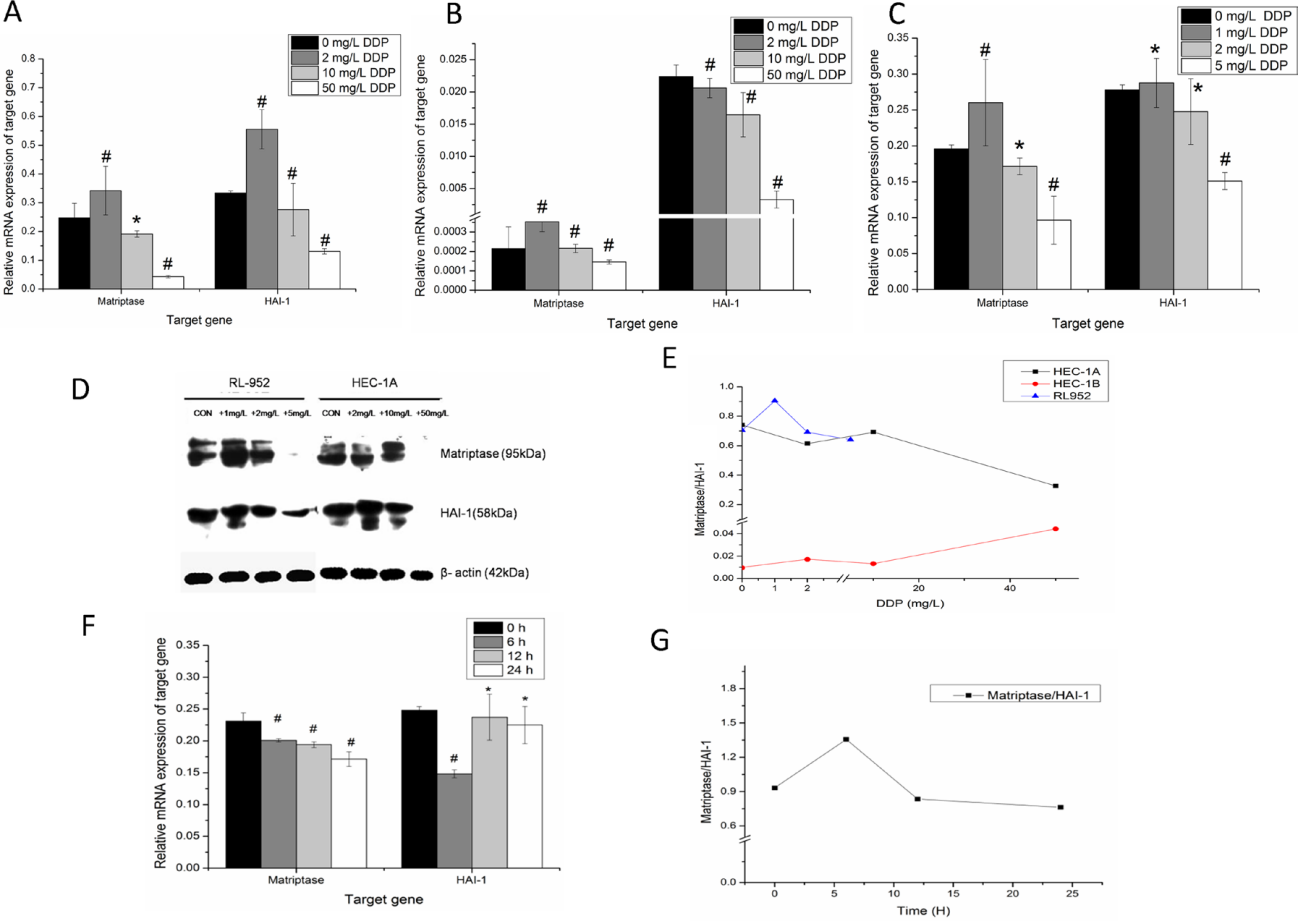
Dose-dependent regulation of matriptase, HAI-1 by cisplatin in endometrial cancer cells After treatment with different concentration of cisplatin for 24 h, down regulation of the matriptase and HAI-1 mRNA were observed in endometrial cancer cells with increasing dose of cisplatin in HEC-1A (**A**), HEC-1B (**B**) and RL-952 (**C**). (**D**) The western-blot analysis for the protein expression of matriptase and HAI-1 show similarly trend with mRNA expression after cisplatin treatment. (**E**) The ratio of matriptase/HAI-1 was initially raised and then decreased at the increased cisplatin concentration in HEC-1A and RL-952 cells. (**F**) After treated RL-952 cell with 2 mg/L DDP cisplatin from 0h to 24 h, there was obvious time-dependent manner between cisplatin and matriptase mRNA. (**G**) The ratio of matriptase/HAI-1 at specific time point after treated with cisplatin. ^*^P < 0.05, ^#^*P* > 0.05.

After treatment with cisplatin at different concentrations for 24 h, the expression levels of matriptase and HAI-1 mRNA significantly changed in all three cell lines (*P* < 0.005) (Figure 4A–4C). Detected by quantitative-PCR, the relatively matriptase mRNA levels were 0.247 ± 0.050, 0.342 ± 0.084, 0.191 ± 0.011, 0.043 ± 0.004 and HAI-1 mRNA levels were 0.334 ± 0.008, 0.555 ± 0.068, 0.276 ± 0.090, 0.131 ± 0.009 (all *P* < 0.001) in HEC-1A cells treated with 0 mg/L, 2 mg/L, 10 mg/L, and 50 mg/L cisplatin, respectively. Although matriptase and HAI-1 were observed very weak expression in HEC-1B cells, the data were also analyzed in cells treated with 0 mg/L, 2 mg/L, 10 mg/L, and 50 mg/L cisplatin. For, matriptase, they were 2.1E-4 ± 1.1E-4, 3.5E-4 ± 5.1E-5, 2.2E-4 ± 2.1E-5, 1.4E-4 ± 1.1E-5 and for HAI-1, they were 0.022 ± 0.001, 0.021 ± 0.001, 0.016 ± 0.003, 0.003 ± 0.001 (all *P* < 0.001), respectively. Compared with HEC-1A and HEC-1b cells, the RL-952 cell line is more sensitive to the treatment with cisplatin. After treated with 0 mg/L, 1 mg/L, 2 mg/L and 5 mg/L cisplatin, the matriptase mRNA in RL-952 cells were 0.196 ± 0.005, 0.260 ± 0.060, 0.171 ± 0.012, 0.097 ± 0.033 (*P* < 0.005) and HAI-1 mRNA were 0.278 ± 0.007, 0.288 ± 0.034, 0.248 ± 0.046, 0.151 ± 0.011 (*P* < 0.005) respectively. The maximum inhibition of matriptase and HAI-1 mRNA expression levels was observed in HEC-1A and in RL-952 cells treated with 50 mg/L and 5 mg/L cisplatin, respectively (Figure 4A, 4B). However, when the cells were treated with cisplatin at low concentration, the expression levels of matriptase and HAI-1 in both HEC-1A and RL-952 groups were higher than when cells were treated at high cisplatin concentration. Western blot analysis showed a similar result for the protein expression of both matriptase and HAI-1 (Figure 4B). This study indicated that both matriptase and HAI-1 mRNA expression levels were cisplatin dose-dependent. The ratio of matriptase/HAI-1 mRNA initially raised and then decreased at increasing cisplatin concentration in both HEC-1A and RL-952 cells (Figure 4E). Additionally, the time correlation between cisplatin incubation and matriptase and HAI-1 mRNA expression was monitored using RL-952 as the cellular model. The data recorded at different time-points (0, 6, 12, and 24 h) showed obvious dependence of matriptase mRNA expression levels and cisplatin incubation time (*P* = 0.01) (Figure 4F). Moreover, upon one-way ANOVA of the data, we could find the statistical significance in the HAI-1 mRNA between the experimental groups (0 h and 6 h). Furthermore, after an initial increase, the matriptase/HAI-1 ratio decreased with increasing cisplatin incubation time (Figure 4G).

### Effect of cisplatin on migration and invasion of endometrial cancer cells by regulation of matriptase and HAI-1 expression

**Figure 5:**
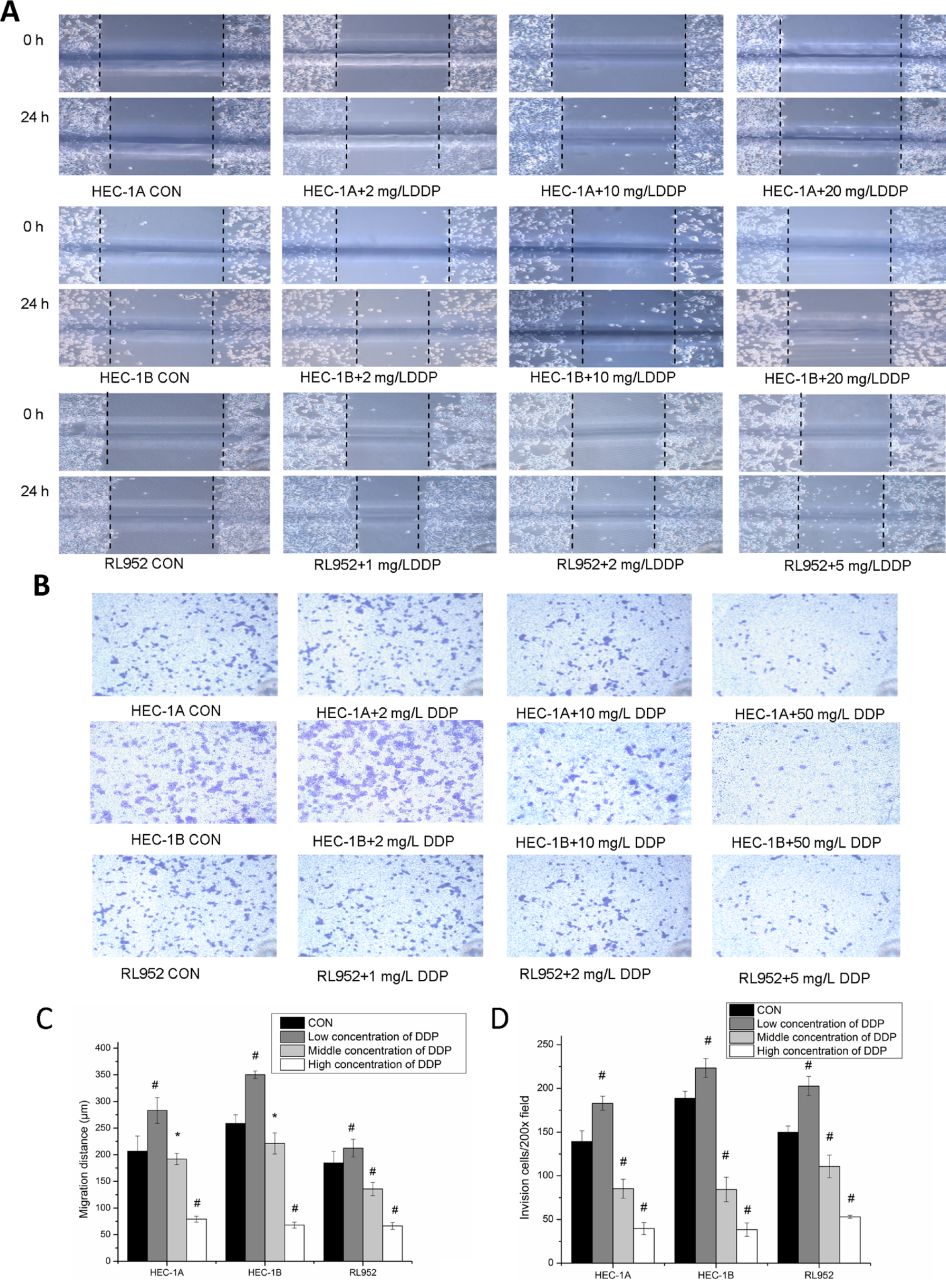
Inhibition of metastatic ability of endometrial cancer cells via matriptase suppression by cisplatin (**A**) After treated by different concentrations of cisplatin, migration were measured by scratch assays, magnification, x200. (**B**) After treated by different concentrations of cisplatin, invasion ability was measured by Transwell assays, magnification, x200. (**C**) the migration distance of three cell lines shows similar change trend with expressions of matriptase and HAI-1. migration distance of endometrial cancer cell in low concentrations cisplatin group was widened compared with blank control group; and migration distances were significantly reduced with the increase concentration of cisplatin. (**D**) The number of endometrial cancer cells that migrated through the Transwell membrane was increased in low concentrations cisplatin group compared with blank control group; and transmembrane cell number was significantly reduced with the increase concentration of cisplatin. ^*^mean *P* < 0.05, ^#^mean *P* > 0.05.

Observation of the migration distance in the three cell lines revealed consistent changes among all the groups studied. Particularly, the migration distance of cells in groups treated with low cisplatin concentrations was larger than that in the control group; however, it was significantly reduced at high cisplatin concentration (*P* < 0.001) (Figure 5A). The 24-h cell migration distances were (206.67 ± 28.28) μm, (283.25 ± 24.19) μm, (192 ± 10.58) μm, and (79.2 ± 5.40) μm respectively in HEC-1A cells treated with 0 mg/L, 2 mg/L, 10 mg/L, and 50 mg/L cisplatin, respectively; (258.67 ± 26.23) μm, (350.44 ± 6.93) μm, (181.2 ± 19.8) μm, and (34.67 ± 5.54) μm in HEC-1B cells treated with 0 mg/L, 2 mg/L, 10 mg/L, and 50 mg/L cisplatin, respectively; and (184.57 ± 21.97) μm, (212.23 ± 16.58) μm, (135.78 ± 12.36) μm, and (66.4 ± 6.56) μm in RL-952 cells treated with 0 mg/L, 1 mg/L, 2 mg/L and 5 mg/L cisplatin, respectively. The differences between the various concentrations groups in the three cell lines were all statistically significant (P_HEC-1A_ < 0.001; P_HEC-1B_ < 0.001; P _RL-952_ < 0.001) (Figure 5C). In agree with the change of matriptase and HAI-1 mRNA, the migration and invasion of endometrial cancer cells showed identical alternation. Pearson's correlation analysis of the migration distance and matriptase and HAI-1 expression levels in the three endometrial carcinoma cell lines treated with various cisplatin concentrations showed strong correlation (matriptase: *r* = 0.873, 0.743, and 0.824 in HEC-1A, HEC-1B, and RL-952 cells, respectively, *P* < 0.01; HAI-1: *r* = 0.934, 0.830, and 0.824 in HEC-1A, HEC-1B, and RL-952 cells, respectively, *P* < 0.01).

The results obtained with the invasion assay were consistent with those obtained with the scratches experiment (Figure 5B). Transmembrane cell counts in HEC-1A cells treated with 0, 2, 10, and 50 mg/L cisplatin were 139.25 ± 12.31, 183 ± 8.16, 85.25 ± 10.87, and 39.75 ± 6.85, respectively (F = 168.184, *P* < 0.001). Transmembrane cell counts in HEC-1B cells treated with 0, 2, 10, and 50 mg/L cisplatin were 188.67 ± 8.08, 223.33 ± 10.69, 84.33 ± 14.01, and 38.33 ± 7.64, respectively (F = 208.018, *P* < 0.001; *P* < 0.01 between each concentration group). Transmembrane cell counts in RL-952 cells treated with 0, 1, 2, and 5 mg/L cisplatin concentrations were 150 ± 7.07, 202.67 ± 11.15, 110.75 ± 12.97, and 53 ± 1.73, respectively (F = 99.966, *P* < 0.001; *P* < 0.01 between each concentration group) (Figure 5D). Pearson's correlation analysis of the transmembrane cell number in the three endometrial carcinoma cells at various cisplatin doses showed strong correlation with matriptase (*r* = 0.874, 0.642, and 0.887 in HEC-1A, HEC-1B, and RL-952 cells, respectively; *P* < 0.01) and HAI-1 expression levels (*r* = 0 928, 0.844, and 0.779 in HEC-1A, HEC-1B, and RL-952 cells, respectively; *P* < 0.01).

### Effect of cisplatin on endometrial cancer cell survival rate

**Figure 6:**
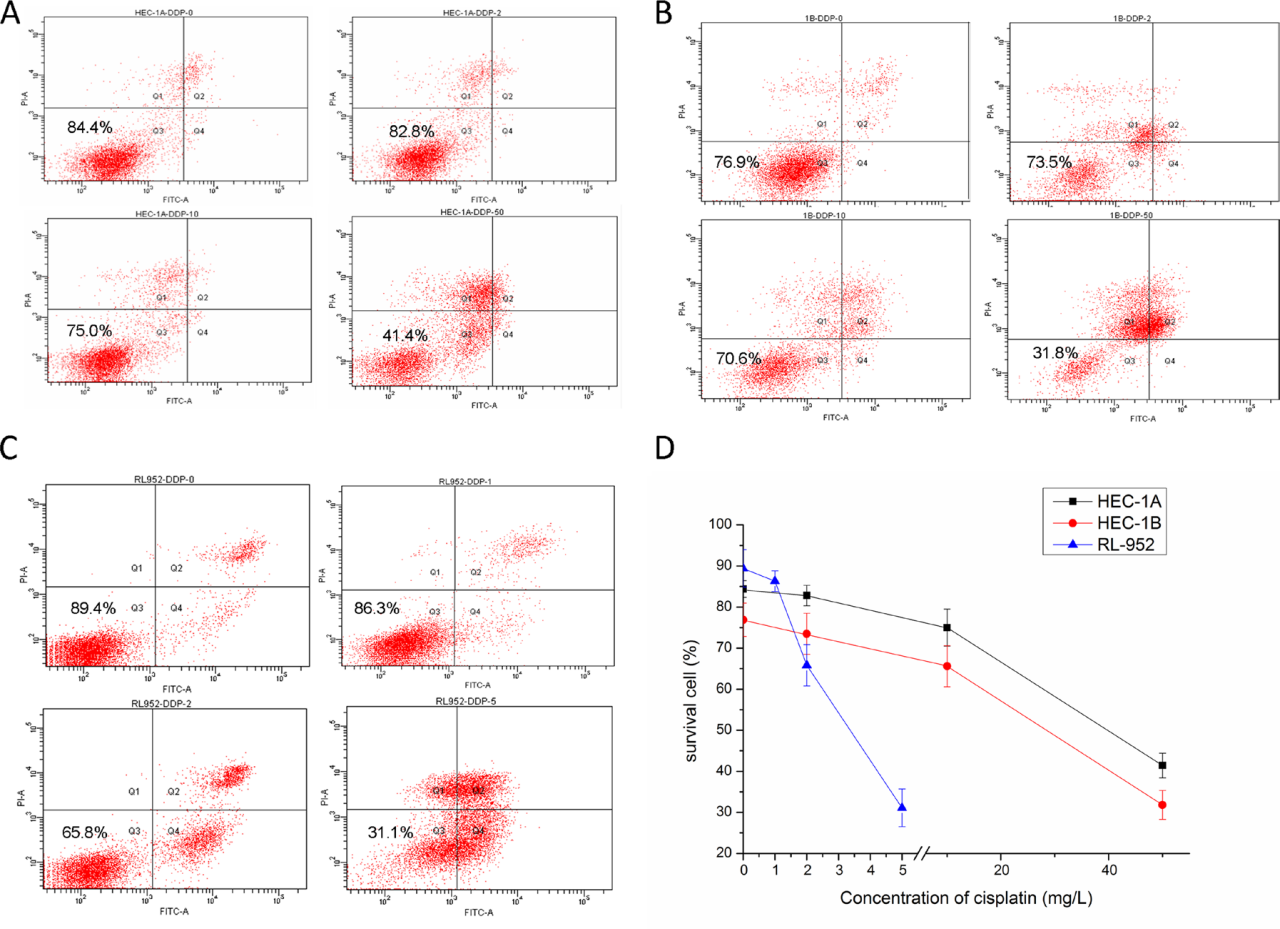
Effect of cisplatin on endometrial cancer cell survival rate The result of flow cytometry after treated with cisplatin in HEC-1A (**A**), HEC-1B (**B**), RL952 (**C**) cell respectively. (**D**) The survival rate of HEC-1A, HEC-1B, RL952 cells were decreased with the increasing concentration of cisplatin.

Cell survival rates decreased with increasing cisplatin concentration in all the three cell lines (Figure 6). The survival rates of the HEC-1A cell groups treated with 0, 2, 10, and 50 mg/L cisplatin were 84.4%, 82.8%, 75.0%, and 41.4%, respectively (Figure 6A). Additionally, the survival rates of the HEC-1B cell groups treated with 0, 2, 10, and 50 mg/L cisplatin were 76.9%, 73.5%, 65.6%, and 31.8%, respectively (Figure 6B). Finally, the survival rates of the RL-952 cell groups treated with 0, 1, 2, and 5 mg/L cisplatin were 89.4%, 86.3%, 65.8%, and 31.1%, respectively (Figure 6C). The difference among the different concentration groups for each cell type was statistically significant (*P* < 0.001). With all the cell types, the survival rate had a consistent trend, with the groups treated with low cisplatin concentration having slightly lower rates than that of the corresponding control group (*P* > 0.05). Person's correlation analysis demonstrated that the cell survival rate was positively correlated with matriptase mRNA (*r* = 0.819, *r* = 0.512, and r = 0.783 in HEC-1A, HEC -1B, and RL-952 cells, respectively; *P* < 0.01) and HAI-1 expression levels (*r* = 0.711, r = 0.964, and *r* = 0.866 in HEC-1A, HEC -1B, and RL-952 cell, respectively; *P* < 0.01).

## DISCUSSION

To date, there are no effective therapeutic options for patients with advanced-stage or recurrent endometrial cancer. Despite many efforts, this disease inevitably progresses with great morbidity, to eventually cause death. Matriptase and its inhibitor HAI-1, which may play a central role in the “success” of metastatic deposits, are interesting candidates for endometrial cancer therapy. However, until recently, only a few published studies attempted to address the importance of matriptase and HAI-1 in endometrial cancer cells. Here, we detected both the mRNA and protein expression of matriptase and HAI-1 in three endometrial cancer cell lines, i.e., HEC-1A, HEC-1B, and RL-952. The results of this study showed that RL-952 and HEC-1A cells are characterized by positive expression of matriptase and HAI-1, while HEC-1B cells display extreme low expression of the matriptase/HAI. We speculated that these data might relate to the different cell's genetic profile such as the expression of the estrogen receptor. Some studies concluded that matriptase mRNA levels were not significantly increased in breast cancer compared to in normal breast tissue, but other studies reported that high matriptase expression is predictive of poor survival for breast cancer as assessed by immunohistochemistry [20, 21]. The process of invasion and metastasis of malignant tumor is complex and it may involve various signaling pathways such as those linked to protease-activated receptors [22], prostasin [23], and micro-RNAs [24]. Several reports suggested that the expression of matriptase is involved in the initiation of malignant progression in epithelial cell carcinogenesis and indicated the potential value of matriptase as prognostic marker in various human cancers [11, 25, 26]. Moreover, matriptase may play an important role in cell invasiveness and metastasis [27, 28]. Nakamura et al. reported that the expression of matriptase in endometrial cancer may be associated with aggressive biological characteristics and may play an important role in prognosis and/or recurrence [18]. Interestingly, strong matriptase expression at both the mRNA and protein level was associated with high metastatic ability and invasiveness [17]. Therefore, we targeted suppressed matriptase expression in HEC-1A and RL-952 cells using lentivirus-mediated siRNA. Real-time qualitative PCR and western blotting revealed that siRNA transfection significantly decreased matriptase expression in both cells (*P* < 0.01), resulting in significant decreases of the cell invasiveness and migratory activity.

The expression of HAI-1, which has an important role in ECM degradation in endometrial cancer, was obviously lower than that in normal endometrium specimens. Increased expression levels of the HAI-1 gene accompanied with decreased expression of matriptase inhibits endometrial cancer cell proliferation and invasive migration [19]. Some scholars have suggested that maintaining matriptase/HAI-1 equilibrium is crucial in the process of tumor progression. According to previous reports, matriptase/HAI-1 ratio imbalance, which includes increased expression of matriptase or decreased expression of HAI-1, may promote tumor development. In pancreatic cancer cells, increased matriptase/HAI-1 ratio by decreased HAI-1 expression levels promoted tumor invasion [29]. Similarly, increased matriptase/HAI-1 ratio with increased matriptase expression made prostate cancer more aggressive [14, 25]. The matriptase/HAI-1 ratio declined in advanced colorectal cancer and infiltrating breast cancer, while it increased in invasive breast cancer [30, 31]. To our knowledge, this is the first study reporting on the regulation of the matriptase/HAI-1 ratio by specific (i.e., siRNA) and non-specific (i.e., cisplatin) targeting associated with decreasing matriptase expression in endometrial cancer cells. In our previous study, we concluded that the expression of matriptase is directly and positively correlated with ovarian cellular invasion and metastasis [17]. Usage of siRNA specifically reduced matriptase expression in matriptase/HAI-1-positive RL-952 and HEC-1A cells. Although HAI-1 gene expression did not change, matriptase/HAI-1 ratio decreased from 0.77 to 0.11 after the first knockdown, and cell invasion and migration decreased significantly.

Since platinum-based combination chemotherapy is currently the main therapy for endometrial cancer, in this study, we treated endometrial cancer cell lines with different cisplatin concentrations. The results showed that invasiveness, metastasis, and apoptosis of matriptase/HAI-1-positive HEC-1A and RL-952 endometrial cancer cells after treatment with normal therapeutic concentration or higher cisplatin doses are positively related to the expression of matriptase and HAI-1. Interestingly, when the cells were treated with low cisplatin concentration, both HEC-1A and RL-952 cells showed increased expression of matriptase and HAI-1, which resulted in enhanced invasion and migration of endometrial cancer cells. Liu *et al.* [32] reported that short-time low cisplatin concentration treatment leads to elevated invasiveness of prostate cancer cell *in vitro*, which is possibly due to epithelial mesenchymal transition (EMT). In addition, Chen *et al.* [33] demonstrated that low-dose cisplatin contributes to the development of drug resistance to promote proliferation of ovarian cancer. The above findings provide novel insights into the mechanisms underlying how low cisplatin concentration treatment leads to elevated tumor progression. Since too low cisplatin doses do not induce tumor cell apoptosis and increased expression of matriptase and HAI-1, these could be clinical indexes for the determination of whether cisplatin doses were administered at effective therapeutic concentrations.

In summary, our findings suggest that the mRNA and protein levels of matriptase and HAI-1 are reliable biomarkers that reflect the aggressive nature of endometrial cancer cells. Matriptase and HAI-1 are potential therapeutic targets for the inhibition of endometrial cancer invasion and metastasis, and could be used as indicators of the curative effect of cisplatin.

## MATERIALS AND METHODS

### Cell lines and cell culture

Endometrial cancer cell lines HEC-1A, HEC-1B, and RL-952 were purchased from the American Typical Culture Collection (ATCC, Rockville, USA). HEC-1A and HEC-1B were cultured in 90% Dulbecco's modified Eagle's medium (DMEM, Gibco, Carlsbad, USA), supplemented with 10% fetal bovine serum (FBS, Gibco), 4 mM L-glutamine (Sigma, Louis, USA), 4.5 g/L glucose, 1.0 mM sodium pyruvate, 1% penicillin (100 IU/mL), and 1% streptomycin (100 IU/mL) in a 5%-CO_2_ incubator at 37°C. RL-952 was cultured in 90% DMEM/F12 1:1 medium (Gibco) supplemented with 10% FBS (Gibco), 1% penicillin (100 IU/mL) and 1% streptomycin (100 IU/mL) in a 5%-CO_2_ incubator at 37°C.

### Reverse transcription polymerase chain reaction

As we previously described [17], total RNA was isolated according to the manufacturer's protocol (Invitrogen, USA, Thermal). The quality and content of mRNA were assessed using a DNA Counter NanoDrop2000 (Thermal). Only samples with an optical density (OD) 260/280 ratio exceeding 1.8 were used in the experiments. The mRNA was transcribed into cDNA using an Access Real-time PCR system (Promega, USA). The following primer sets were synthesized by Sanggong Biotech (Shanghai, China). For matriptase, sense 5′-GGG ACA CAC CCA GTA TGG AGG-3′ and anti-sense 5′-CCG GAA TCA CCC TGG CAG GA-3′ (168 bp); for HAI-1: sense 5′- GGC AAC AAG AAC AAC TTT GAG GA-3′ and anti-sense 5′- CAA TGC AGA TGA CCA GGA ACA -3′ (154 bp); for GAPDH, sense 5′- GAA GGT GAA GGT CGG AGT C-3′, anti-sense: 5′- GAA GAT GGT GAT GGG ATT TC -3′ (226 bp). The program for RT-PCR was as follows: 95°C for 15 s, 45 cycles of denaturation at 95°C for 5 s and annealing at 60°C for 20 s, 95°C for 1 min, and cooling to 55°C. the PCR products were quantified by agarose gel electrophoresis. DNA marker was 600bp (Sanggong Biotech, Shanghai, China).

### Fluorescence Real-time quantitative PCR

Real-time quantitative PCR was performed by using a LightCycler^®^ 480 SYBR Green I Master Mix (Roche, Germany). The following primer sets were synthesized by Sanggong Biotech (Shanghai, China). For matriptase, sense 5′-TCG TCA CTT GTA CCA AAC ACA CCT A-3′ and anti-sense 5′-GAG CCT GTC TCG TGA ATG ACC-3′ (150 bp); for HAI-1: sense 5′- GGC AAC AAG AAC AAC TTT GAG GA-3′ and anti-sense 5′-CAA TGC AGA TGA CCA GGA ACAC-3′ (154 bp); for GAPDH, sense 5′- GCACCGTCAAGGCTGAGAAC-3′, anti-sense: 5′- TGGTGAAGACGCCAGTGGA-3′ (138 bp) [17]. The program for real-time PCR was as follows: 95°C for 15 s, 45 cycles of denaturation at 95°C for 5 s and annealing at 60°C for 20 s, 95°C for 1 min, and cooling to 55°C. The relative mRNA levels were calculated using the comparative cycle threshold (C_t_) method (ΔΔC_t_). Briefly, the C_t_ value for GADPH was subtracted from the C_t_ value of the target gene to achieve the ΔC_t_ value. The 2^−ΔCt^ value was calculated for each sample and each value was then divided by that of the control to determine the relative mRNA levels (ΔΔCt).

### Western blotting

Cells were plated at a density of 3 × 10^5^ cells/well in 35-mm plates. As previously described [17], whole-cell proteins were extracted according to the manufacturer's protocol (Clontech, Palo Alto, USA) and determined by enzyme-linked immunosorbent assay (Pierce). Precisely, 100 μg of whole-cell protein was loaded per lane on an 8%-polyacrylamide gel. Proteins were blotted onto nitrocellulose membranes. The blots were washed in PBS and incubated in blocking buffer (1× PBS, 0.1% Tween-20, 5% I-Block) at 20°C for 1 h. Membranes were incubated overnight at 20°C with rabbit polyclonal antibody specific to matriptase (1: 1,000 dilution, ab28266, Abcam) or rabbit monoclonal antibody specific to HAI-1 (1: 2,000 dilution, ab189511, Abcam) in blocking buffer, followed by incubation with an alkaline phosphatase-conjugated anti-rabbit secondary antibody (1:1000 dilution, BA1054, Boster, Wuhan, China). Bands were visualized using the CDP star RTU luminescence system (Tropix).

### Lentivirus-mediated small interfering RNA construction and infection

Three lentivirus-mediated small interfering RNA constructs were constructed and named siRNA-Ma-1–3. The following siRNA target sequences in the matriptase gene (ST-14, GenBank accession No. NM_021978) were selected: Ma-SiRNA-1, CCGGCTTCTTAGCTGAATA; Ma-SiRNA-2, TGTCCAGAAGGTCTTCAAT; and Ma-SiRNA-3, ACGAGAAAGTGGAATGGCTT [17]. Then, three pairs of complementary oligonucleotides were designed, and stem-loop oligonucleotides were synthesized and cloned into a lentivirus-based vector carrying the green fluorescent protein (GFP) gene (GV115, Genechem, Shanghai, China). A universal sequence (PSC-NC: TTCTCCGAACGTGTCACGT, named NC) was used as the negative control for RNA interference. Lentiviral particles were prepared as previously described [34]. Three Ma-siRNA-carrying and NC-carrying lentiviral vectors were constructed and infected into endometrial cancer cells each at multiplicities of infection (MOIs) of 20 (low MOI) and 80 (high MOI). After infection for 72 h, GFP expression was detected to calculate the infection efficiency. Five days after infection, cells were harvested. Real-time PCR was performed to determine the efficiency of matriptase depletion and screen for the siRNA with the highest silencing efficiency, which was then used for subsequent experiments. Three groups of cells were set: cells infected with lentivirus-mediated siRNA target on matriptase (group KD), cells infected empty lentivirus (group NC), and cells treat with DMSO as the control (group CON).

### *In vitro* cellular scratch test

Cells, either treated with drug or siRNA, were allowed to grow to confluence in 6-well plates. A 200-μL tip was used to introduce a scratch in the monolayer. The scratch areas in wells were washed with PBS and 1 mmol/L *R*-flurbiprofen until the cells in those areas were removed thoroughly. The wells were imaged at ×40 magnification with an Olympus IX70 inverted-fluorescence microscope (Olympus, Japan) at 0 and 24 h post-scratching. Scratch healing was determined by measuring the shortest distance between scratch edges in each field of view. Three different fields were measured per scratch, and 20 different measurements were taken per field. The distance between scratches was measured using the Image ProExpress C software (Olympus, Japan) and the horizontal migration rate was calculated using the following formula: (width_0 h_ − width_24 h_)/width_0 h_ × 100% [17, 35].

### Trans-well chamber migration assay

After thawing overnight at 4°C on ice, 50 μL of Matrigel™ Basement Membrane Matrix (BD, USA) was added to a Millicell Hanging Cell Culture Insert (Millipore, USA) to coat the membrane and incubated at 37°C for 30 min. The membrane was re-hydrated with FBS-free DMEM thrice. Cells in the logarithmic growth phase were suspended in the purpose medium containing 0.5% FBS after conventional digestion. Then, 200-μL cell suspensions (5.0 × 10^5^/mL, untreated with drug or siRNA for 72 h) were added to the Hanging Cell Culture Insert placed in 24-well places containing 1,300μL of DMEM supplemented with 10% FBS. The plates were incubated for 24 h at 37°C. At the end of incubation, non-migrating cells on the inside of the filter were removed with a cotton swab, and the filters were fixed with methanol and stained with crystalline violet for 15 min. The filters were removed from the inserts and mounted onto slides for imaging and quantification. The number of migrating cells on the underside of the filter was determined by counting cells in 5 random fields from 3 filters for each treatment at ×200 magnification using an inverted microscope (Olympus) [17, 36].

### Treatment with cisplatin

Cisplatin (Sigma) was prepared in 100% dimethylsulphoxide (DMSO). Before drug treatment, endometrial cancer cells were seeded in 6-well plates at a density of 1 × 10^5^ cells per well and cultured in 1 mL of serum-free DMEM for 12 h to achieve adherence. For each cell line, four groups were set. Three groups were treated with cisplatin at different final cisplatin concentration (i.e., 2 mg/L, 10 mg/L, and 50 mg/L for HEC-1A and HEC-1B; and 1 mg/L, 2 mg/L and 5 mg/L for RL-952), and one group was treated with DMSO alone as the control. At different checkpoint times (between 0 and 24 h), Real-time PCR analysis and western blot were performed on 3 wells in RL-952 group per point.

### Cellular survival rate analysis via flow cytometry

For flow cytometric analysis, cells were seeded onto 6-well plates and cultured to 80% confluence. After harvesting, the cell pellets were washed twice with pre-cooled PBS and fixed with pre-cooled 70% ethanol. Then, suspended cells were filtered through a 400-mesh sieve and stained with propidium iodide (PI, 100 μg/mL RNase in PBS) at 37°C for 30 min. Then, the cell cycle distribution was determined using flow cytometry as previously described [37]. Apoptosis was detected using the Annexin-V-FLUOS staining kit (Roche, USA) according to the manufacturer's instructions. Fluorescein and PI fluorescence was measured using a FACSCanto II flow cytometer (BD, USA).

### Statistical analysis

All experiments were performed in triplicate. Statistical analysis was performed using the average results of three experiments under identical conditions. Numerical data are presented as the mean ± SD. Differences between two means were compared by Student's *t*-test, and related parameters were analyzed using Pearson's correlation analysis. Data were analyzed using SPSS 17.0 for Windows (SPSS Inc., Chicago, IL, USA). Differences were considered significant at *P* < 0.05.
